# (*E*)-3-[4-(Dimethyl­amino)phen­yl]-1-(4-methyl­phen­yl)prop-2-en-1-one

**DOI:** 10.1107/S1600536809052398

**Published:** 2009-12-16

**Authors:** Lei Wang, Li-Ying Ma, Yu-Ling Huang, Bai-Yu Zheng

**Affiliations:** a Binzhou Medical University, Yantai 264003, People’s Republic of China

## Abstract

In the title compound, C_18_H_19_NO, the dihedral angle between 4-methyl­phenyl and 4-(dimethyl­amino)phenyl rings is 45.5 (3)°. The C—C=C—C torsion angle of 173.8 (3)° indicates that the mol­ecule adopts an *E* configuration. The dimethyl­amino group is nearly coplanar with the attached benzene ring, making a dihedral angle of 2.7 (3)°. Weak inter­molecular C—H⋯π inter­actions are observed in the crystal structure.

## Related literature

The title compound is a chalcone derivative; for the biological activiy of chalcones, see: Modzelewska *et al.* (2006 ▶); Opletalova & Sedivy (1999 ▶); Lin *et al.* (2002 ▶); Hsieh *et al.* (1998 ▶); Lunardi *et al.* (2003 ▶); Tang *et al.* (2008 ▶). For the organic non-linear optical properties of chalcones, see: Indira *et al.* (2002 ▶); Ravindra *et al.* (2009 ▶). For related structures, see: Wang *et al.* (2004 ▶); Yang *et al.* (2006 ▶).
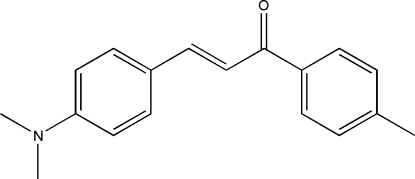


## Experimental

### 

#### Crystal data

C_18_H_19_NO
*M* *_r_* = 265.34
Orthorhombic, 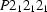

*a* = 7.276 (2) Å
*b* = 11.567 (3) Å
*c* = 17.642 (5) Å
*V* = 1484.8 (7) Å^3^
*Z* = 4
Mo *K*α radiation
μ = 0.07 mm^−1^
*T* = 193 K
0.59 × 0.35 × 0.18 mm

#### Data collection

Rigaku Mercury diffractometer
16704 measured reflections
1958 independent reflections
1846 reflections with *I* > 2σ(*I*)
*R*_int_ = 0.055

#### Refinement

*R*[*F* ^2^ > 2σ(*F* ^2^)] = 0.061
*wR*(*F* ^2^) = 0.136
*S* = 1.31
1958 reflections
185 parameters
H-atom parameters constrained
Δρ_max_ = 0.18 e Å^−3^
Δρ_min_ = −0.18 e Å^−3^

### 

Data collection: *CrystalClear* (Rigaku, 1999 ▶); cell refinement: *CrystalClear*; data reduction: *CrystalStructure* (Rigaku, 2000 ▶); program(s) used to solve structure: *SHELXS97* (Sheldrick, 2008 ▶); program(s) used to refine structure: *SHELXL97* (Sheldrick, 2008 ▶); molecular graphics: *SHELXTL* (Sheldrick, 2008 ▶); software used to prepare material for publication: *SHELXL97*.

## Supplementary Material

Crystal structure: contains datablocks I, global. DOI: 10.1107/S1600536809052398/xu2690sup1.cif

Structure factors: contains datablocks I. DOI: 10.1107/S1600536809052398/xu2690Isup2.hkl

Additional supplementary materials:  crystallographic information; 3D view; checkCIF report

## Figures and Tables

**Table 1:** Hydrogen-bond geometry (Å, °) *Cg*1 and *Cg*2 are the centroids of the C1–C6 and C7–C11 rings, respectively.

*D*—H⋯*A*	*D*—H	H⋯*A*	*D*⋯*A*	*D*—H⋯*A*
C11—H11⋯*Cg*1^i^	0.95	2.94	3.697 (3)	138
C9—H9⋯*Cg*2^ii^	0.95	2.93	3.712 (3)	141
C16—H16*B*⋯*Cg*2^iii^	0.98	2.70	3.643 (3)	161

Symmetry codes: (i) 
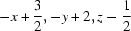
; (ii) 
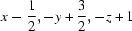
; (iii) 
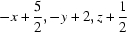
.

**Fig. 1.**
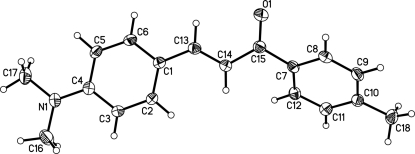
The molecular structure of (I), with 30% probability displacement ellipsoids and atom labels for non-H atoms.

**Fig. 2.**
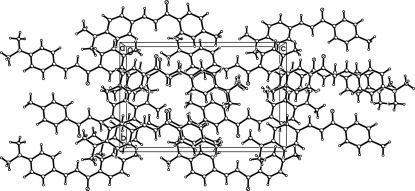
The packing of (I), viewed down the a axis.

## supplementary crystallographic information

### Comment

Chalcones are open chain flavonoids which consist of two substituted benzene rings bridged by a prop-2-en-1-one group. They are renowned for their broad biological activities (Opletalova & Sedivy, 1999), such as anticancer (Modzelewska *et al.*, 2006), antitubercular (Lin *et al.*, 2002), anti-inflammatory (Hsieh *et al.*, 1998), trypanocidal (Lunardi *et al.*, 2003) and antibacterial properties (Tang *et al.* 2008). In addition, chalcones are also finding applications as organic non-linear optical (NLO) materials for their excellent blue light transmittance and good crystal stabitily (Indira *et al.*, 2002; Ravindra *et al.*, 2009). As a part of our searches for NLO materials based on chalcones (Wang, *et al.*, 2004; Yang, *et al.*, 2006), the title compound (I) was synthesized and its crystal structure is reported. The crystal should exhibit second-order NLO properties, because it crystallizes in a non-centrosymmetric space group.

In the title compound(I) (Fig.1), the molecule adopts an *E* configuration with respect to C13═C14 double bond [1.340 (4) Å]: the torsion angle C1—C13—C14—C15=173.8 (3)°. The dihedral angle between the C1—C6 ring (Plane A) and the C7—C12 ring (Plane B) is 45.5 (3)°, showing the two phenyl rings are rotated oppositely with respect to the enone segment. The mean plane of C1—C13=C14—C15 (Plane C) makes dihedral angles of 8.7 (3)° and 36.7 (4)° with plane A and plane B, respectively. The phenone O1 atom deviates from plane C by 0.240 (3) Å, suggesting C=O is not coplanar with Plane C. The dimethylamino group (Plane D) is nearly coplanar with the phenyl ring to which it is bound. The dehedral angel beween plane A and plane D is 2.7 (3)°. While no classical hydrogen bonds are present, weak intermolecular C—H···π interactions are observed, which contribute to the stability of crystal packing (Table 1).

### Experimental

The synthesis of the title compound was carried out by adding an aqueous solution of sodium hydroxide (10%, 10 ml) to a solution of 4-methylacetophenone (0.02 mol) and 4-(dimethylamino)benzaldehyde (0.02 mol). The reaction mixture was stirred for 5 h at room temperature and then neutralized with HCl solution (10%). The product was recrystallized three times from ethanol (95%). Crystals suitable for X-ray analysis were grown by slow evaporation of the acetone solution at room temperature.

### Refinement

All of the H Atoms were placed in their calculated positions and then refined using the riding model with C—H = 0.95–0.98 Å, and with *U*_iso_ (H)=1.2 or 1.5*U*_eq_(C). In the absence of significant anomalous scattering, Friedel pairs were merged.

### Crystal data

**Table:**

C_18_H_19_NO	*F*(000) = 568
*M**_r_* = 265.34	*D*_x_ = 1.187 Mg m^−^^3^
Orthorhombic, *P*2_1_2_1_2_1_	Mo *K*α radiation, λ = 0.71070 Å
Hall symbol: p 2ac 2ab	Cell parameters from 5334 reflections
*a* = 7.276 (2) Å	θ = 3.0–27.5°
*b* = 11.567 (3) Å	µ = 0.07 mm^−^^1^
*c* = 17.642 (5) Å	*T* = 193 K
*V* = 1484.8 (7) Å^3^	Block, yellow
*Z* = 4	0.59 × 0.35 × 0.18 mm

### Data collection

**Table:**

Rigaku Mercury diffractometer	1846 reflections with *I* > 2σ(*I*)
Radiation source: fine-focus sealed tube	*R*_int_ = 0.055
graphite	θ_max_ = 27.5°, θ_min_ = 3.0°
Detector resolution: 7.31 pixels mm^-1^	*h* = −9→9
ω scans	*k* = −14→14
16704 measured reflections	*l* = −19→22
1958 independent reflections	

### Refinement

**Table:**

Refinement on *F*^2^	Primary atom site location: structure-invariant direct methods
Least-squares matrix: full	Secondary atom site location: difference Fourier map
*R*[*F*^2^ > 2σ(*F*^2^)] = 0.061	Hydrogen site location: inferred from neighbouring sites
*wR*(*F*^2^) = 0.136	H-atom parameters constrained
*S* = 1.31	*w* = 1/[σ^2^(*F*_o_^2^) + (0.0495*P*)^2^ + 0.3222*P*] where *P* = (*F*_o_^2^ + 2*F*_c_^2^)/3
1958 reflections	(Δ/σ)_max_ < 0.001
185 parameters	Δρ_max_ = 0.18 e Å^−^^3^
0 restraints	Δρ_min_ = −0.18 e Å^−^^3^

### Special details

**Table:**

Geometry. All e.s.d.'s (except the e.s.d. in the dihedral angle between two l.s. planes) are estimated using the full covariance matrix. The cell e.s.d.'s are taken into account individually in the estimation of e.s.d.'s in distances, angles and torsion angles; correlations between e.s.d.'s in cell parameters are only used when they are defined by crystal symmetry. An approximate (isotropic) treatment of cell e.s.d.'s is used for estimating e.s.d.'s involving l.s. planes.
Refinement. Refinement of *F*^2^ against ALL reflections. The weighted *R*-factor *wR* and goodness of fit *S* are based on *F*^2^, conventional *R*-factors *R* are based on *F*, with *F* set to zero for negative *F*^2^. The threshold expression of *F*^2^ > σ(*F*^2^) is used only for calculating *R*-factors(gt) *etc*. and is not relevant to the choice of reflections for refinement. *R*-factors based on *F*^2^ are statistically about twice as large as those based on *F*, and *R*- factors based on ALL data will be even larger.

### Fractional atomic coordinates and isotropic or equivalent isotropic displacement parameters (Å^2^)

**Table:**

	*x*	*y*	*z*	*U*_iso_*/*U*_eq_	
O1	0.9451 (4)	0.62914 (17)	0.28002 (11)	0.0570 (7)	
N1	1.0052 (4)	0.9449 (2)	−0.14961 (12)	0.0474 (7)	
C1	0.9388 (4)	0.8121 (2)	0.07087 (15)	0.0340 (6)	
C2	1.0071 (4)	0.9237 (2)	0.05857 (14)	0.0342 (6)	
H2	1.0393	0.9701	0.1010	0.041*	
C3	1.0288 (4)	0.9681 (2)	−0.01345 (15)	0.0355 (6)	
H3	1.0754	1.0442	−0.0195	0.043*	
C4	0.9833 (4)	0.9029 (2)	−0.07801 (15)	0.0361 (6)	
C5	0.9100 (4)	0.7916 (2)	−0.06585 (15)	0.0389 (6)	
H5	0.8734	0.7458	−0.1079	0.047*	
C6	0.8909 (4)	0.7489 (3)	0.00638 (17)	0.0377 (6)	
H6	0.8431	0.6732	0.0127	0.045*	
C7	0.9790 (4)	0.7943 (2)	0.35669 (14)	0.0339 (6)	
C8	1.0590 (4)	0.7345 (2)	0.41698 (16)	0.0395 (6)	
H8	1.1025	0.6580	0.4094	0.047*	
C9	1.0757 (4)	0.7849 (2)	0.48706 (16)	0.0424 (7)	
H9	1.1343	0.7435	0.5268	0.051*	
C10	1.0082 (4)	0.8956 (3)	0.50109 (15)	0.0399 (6)	
C11	0.9305 (4)	0.9556 (3)	0.44122 (15)	0.0410 (7)	
H11	0.8854	1.0317	0.4493	0.049*	
C12	0.9174 (4)	0.9066 (2)	0.36960 (15)	0.0371 (6)	
H12	0.8659	0.9499	0.3291	0.045*	
C13	0.9252 (4)	0.7595 (2)	0.14493 (15)	0.0370 (6)	
H13	0.8853	0.6813	0.1455	0.044*	
C14	0.9611 (4)	0.8060 (2)	0.21291 (14)	0.0377 (6)	
H14	0.9896	0.8860	0.2163	0.045*	
C15	0.9573 (4)	0.7358 (2)	0.28210 (15)	0.0382 (6)	
C16	1.0849 (5)	1.0576 (3)	−0.16376 (19)	0.0606 (9)	
H16A	1.2040	1.0631	−0.1381	0.091*	
H16B	1.0025	1.1177	−0.1444	0.091*	
H16C	1.1022	1.0681	−0.2184	0.091*	
C17	0.9558 (6)	0.8769 (3)	−0.21540 (16)	0.0685 (11)	
H17A	0.8250	0.8568	−0.2129	0.103*	
H17B	1.0294	0.8059	−0.2164	0.103*	
H17C	0.9794	0.9218	−0.2615	0.103*	
C18	1.0179 (5)	0.9481 (3)	0.57936 (17)	0.0571 (9)	
H18A	0.9012	0.9352	0.6058	0.086*	
H18B	1.0408	1.0314	0.5751	0.086*	
H18C	1.1179	0.9119	0.6080	0.086*	

### Atomic displacement parameters (Å^2^)

**Table:**

	*U*^11^	*U*^22^	*U*^33^	*U*^12^	*U*^13^	*U*^23^
O1	0.0875 (18)	0.0342 (10)	0.0492 (12)	−0.0048 (12)	0.0013 (13)	0.0026 (9)
N1	0.0567 (17)	0.0519 (14)	0.0335 (12)	−0.0061 (14)	0.0025 (12)	0.0008 (11)
C1	0.0297 (12)	0.0345 (12)	0.0378 (13)	−0.0005 (12)	0.0019 (11)	−0.0026 (11)
C2	0.0345 (13)	0.0343 (13)	0.0339 (13)	0.0001 (12)	−0.0010 (12)	−0.0039 (11)
C3	0.0337 (14)	0.0334 (13)	0.0394 (14)	−0.0019 (11)	−0.0012 (12)	−0.0015 (11)
C4	0.0335 (13)	0.0400 (13)	0.0348 (13)	0.0043 (12)	−0.0008 (12)	−0.0008 (11)
C5	0.0375 (14)	0.0431 (14)	0.0360 (14)	−0.0043 (13)	−0.0030 (12)	−0.0099 (12)
C6	0.0354 (14)	0.0350 (12)	0.0426 (14)	−0.0062 (12)	−0.0010 (12)	−0.0056 (11)
C7	0.0297 (13)	0.0369 (13)	0.0351 (13)	−0.0004 (12)	0.0021 (11)	0.0058 (11)
C8	0.0390 (15)	0.0337 (12)	0.0458 (15)	−0.0025 (13)	0.0022 (13)	0.0076 (12)
C9	0.0421 (15)	0.0443 (15)	0.0409 (14)	−0.0033 (14)	−0.0048 (13)	0.0132 (12)
C10	0.0346 (14)	0.0496 (15)	0.0353 (13)	−0.0105 (13)	0.0030 (12)	0.0029 (12)
C11	0.0370 (14)	0.0441 (15)	0.0418 (15)	0.0014 (13)	0.0039 (13)	−0.0037 (12)
C12	0.0351 (14)	0.0385 (13)	0.0377 (14)	0.0056 (13)	−0.0015 (12)	0.0033 (12)
C13	0.0341 (14)	0.0352 (13)	0.0416 (14)	−0.0020 (12)	0.0035 (12)	0.0000 (11)
C14	0.0420 (15)	0.0344 (13)	0.0367 (14)	−0.0035 (13)	0.0011 (12)	0.0015 (11)
C15	0.0388 (14)	0.0365 (14)	0.0393 (14)	−0.0007 (12)	0.0034 (13)	−0.0003 (12)
C16	0.060 (2)	0.072 (2)	0.0499 (18)	−0.016 (2)	0.0025 (17)	0.0142 (16)
C17	0.086 (3)	0.085 (3)	0.0346 (16)	−0.017 (2)	0.0006 (19)	−0.0049 (16)
C18	0.063 (2)	0.074 (2)	0.0347 (14)	−0.017 (2)	0.0046 (16)	−0.0016 (15)

### Geometric parameters (Å, °)

**Table:**

O1—C15	1.238 (3)	C9—C10	1.393 (4)
N1—C4	1.363 (3)	C9—H9	0.9500
N1—C17	1.448 (4)	C10—C11	1.385 (4)
N1—C16	1.448 (4)	C10—C18	1.510 (4)
C1—C6	1.396 (4)	C11—C12	1.388 (4)
C1—C2	1.400 (4)	C11—H11	0.9500
C1—C13	1.445 (4)	C12—H12	0.9500
C2—C3	1.380 (4)	C13—C14	1.340 (4)
C2—H2	0.9500	C13—H13	0.9500
C3—C4	1.405 (4)	C14—C15	1.466 (4)
C3—H3	0.9500	C14—H14	0.9500
C4—C5	1.410 (4)	C16—H16A	0.9800
C5—C6	1.374 (4)	C16—H16B	0.9800
C5—H5	0.9500	C16—H16C	0.9800
C6—H6	0.9500	C17—H17A	0.9800
C7—C12	1.393 (4)	C17—H17B	0.9800
C7—C8	1.396 (4)	C17—H17C	0.9800
C7—C15	1.488 (4)	C18—H18A	0.9800
C8—C9	1.372 (4)	C18—H18B	0.9800
C8—H8	0.9500	C18—H18C	0.9800
			
C4—N1—C17	121.4 (3)	C10—C11—C12	121.1 (3)
C4—N1—C16	121.8 (2)	C10—C11—H11	119.4
C17—N1—C16	116.8 (2)	C12—C11—H11	119.4
C6—C1—C2	116.4 (2)	C11—C12—C7	120.5 (3)
C6—C1—C13	120.0 (2)	C11—C12—H12	119.7
C2—C1—C13	123.6 (2)	C7—C12—H12	119.7
C3—C2—C1	121.8 (2)	C14—C13—C1	128.8 (2)
C3—C2—H2	119.1	C14—C13—H13	115.6
C1—C2—H2	119.1	C1—C13—H13	115.6
C2—C3—C4	121.3 (2)	C13—C14—C15	121.3 (2)
C2—C3—H3	119.3	C13—C14—H14	119.4
C4—C3—H3	119.3	C15—C14—H14	119.4
N1—C4—C3	122.2 (2)	O1—C15—C14	121.9 (2)
N1—C4—C5	120.7 (2)	O1—C15—C7	119.1 (2)
C3—C4—C5	117.1 (2)	C14—C15—C7	118.9 (2)
C6—C5—C4	120.5 (2)	N1—C16—H16A	109.5
C6—C5—H5	119.8	N1—C16—H16B	109.5
C4—C5—H5	119.8	H16A—C16—H16B	109.5
C5—C6—C1	122.9 (3)	N1—C16—H16C	109.5
C5—C6—H6	118.6	H16A—C16—H16C	109.5
C1—C6—H6	118.6	H16B—C16—H16C	109.5
C12—C7—C8	118.1 (2)	N1—C17—H17A	109.5
C12—C7—C15	122.3 (2)	N1—C17—H17B	109.5
C8—C7—C15	119.5 (2)	H17A—C17—H17B	109.5
C9—C8—C7	120.9 (2)	N1—C17—H17C	109.5
C9—C8—H8	119.6	H17A—C17—H17C	109.5
C7—C8—H8	119.6	H17B—C17—H17C	109.5
C8—C9—C10	121.3 (3)	C10—C18—H18A	109.5
C8—C9—H9	119.4	C10—C18—H18B	109.5
C10—C9—H9	119.4	H18A—C18—H18B	109.5
C11—C10—C9	118.0 (3)	C10—C18—H18C	109.5
C11—C10—C18	121.0 (3)	H18A—C18—H18C	109.5
C9—C10—C18	121.1 (3)	H18B—C18—H18C	109.5
			
C6—C1—C2—C3	−1.1 (4)	C8—C9—C10—C11	−2.7 (4)
C13—C1—C2—C3	175.8 (3)	C8—C9—C10—C18	176.7 (3)
C1—C2—C3—C4	−0.1 (4)	C9—C10—C11—C12	1.0 (4)
C17—N1—C4—C3	−179.3 (3)	C18—C10—C11—C12	−178.4 (3)
C16—N1—C4—C3	3.0 (4)	C10—C11—C12—C7	1.4 (4)
C17—N1—C4—C5	−0.5 (4)	C8—C7—C12—C11	−2.0 (4)
C16—N1—C4—C5	−178.3 (3)	C15—C7—C12—C11	176.2 (3)
C2—C3—C4—N1	−179.5 (3)	C6—C1—C13—C14	−178.7 (3)
C2—C3—C4—C5	1.7 (4)	C2—C1—C13—C14	4.6 (5)
N1—C4—C5—C6	179.0 (3)	C1—C13—C14—C15	−173.8 (3)
C3—C4—C5—C6	−2.2 (4)	C13—C14—C15—O1	9.7 (5)
C4—C5—C6—C1	1.1 (4)	C13—C14—C15—C7	−173.7 (3)
C2—C1—C6—C5	0.6 (4)	C12—C7—C15—O1	−151.9 (3)
C13—C1—C6—C5	−176.4 (3)	C8—C7—C15—O1	26.3 (4)
C12—C7—C8—C9	0.3 (4)	C12—C7—C15—C14	31.4 (4)
C15—C7—C8—C9	−178.0 (3)	C8—C7—C15—C14	−150.4 (3)
C7—C8—C9—C10	2.1 (4)		

### Hydrogen-bond geometry (Å, °)

**Table:**

Cg1 and Cg2 are the centroids of the C1–C6 and C7–C11 rings, respectively.

**Table:**

*D*—H···*A*	*D*—H	H···*A*	*D*···*A*	*D*—H···*A*
C11—H11···Cg1^i^	0.95	2.94	3.697 (3)	138
C9—H9···Cg2^ii^	0.95	2.93	3.712 (3)	141
C16—H16B···Cg2^iii^	0.98	2.70	3.643 (3)	161

Symmetry codes: (i) −*x*+3/2, −*y*+2, *z*−1/2; (ii) *x*−1/2, −*y*+3/2, −*z*+1; (iii) −*x*+5/2, −*y*+2, *z*+1/2.
